# Nanoparticle-allergen interactions mediate human allergic responses: protein corona characterization and cellular responses

**DOI:** 10.1186/s12989-016-0113-0

**Published:** 2016-01-16

**Authors:** Isabella Radauer-Preiml, Ancuela Andosch, Thomas Hawranek, Ursula Luetz-Meindl, Markus Wiederstein, Jutta Horejs-Hoeck, Martin Himly, Matthew Boyles, Albert Duschl

**Affiliations:** 1 Department of Molecular Biology, Division of Allergy and Immunology, University of Salzburg, Hellbrunnerstr, 34, 5020 Salzburg, Austria; 2 Department of Cell Biology, Division of Plant Physiology, University of Salzburg, Salzburg, Austria; 3 Department of Dermatology, Paracelsus Medical University, Salzburg, Austria; 4 Department of Molecular Biology, Division of Structural Biology and Bioinformatics, University of Salzburg, Salzburg, Austria; 5 Heriot-Watt University, Edinburgh, UK

**Keywords:** Gold nanoparticles, Protein corona, Allergy, Basophil activation, Protease activity, A549 cells

## Abstract

**Background:**

Engineered nanomaterials (ENMs) interact with different biomolecules as soon as they are in contact, resulting in the formation of a biomolecule ‘corona’. Hence, the ‘corona’ defines the biological identity of the ENMs and could affect the response of the immune system to ENM exposure. With up to 40 % of the world population suffering from type I allergy, a possible modulation of allergen effects by binding to ENMs is highly relevant with respect to work place and consumer safety. Therefore, the aim of this present study was to gain an insight into the interactions of gold nanoparticles with different seasonally and perennially occurring outdoor and indoor allergens.

**Methods:**

Gold nanoparticles (AuNPs) were conjugated with the major allergens of birch pollen (Bet v 1), timothy grass pollen (Phl p 5) and house dust mite (Der p 1). The AuNP-allergen conjugates were characterized by means of TEM negative staining, dynamic light scattering (DLS), z-potential measurements and hyperspectral imaging. Furthermore, 3D models were constructed, based on the characterization data, to visualize the interaction between the allergens and the AuNPs surface. Differences in the activation of human basophil cells derived from birch/grass pollen- and house dust mite-allergic patients in response to free allergen and AuNP-allergen conjugates were determined using the basophil activation assay (BAT). Potential allergen corona replacement during BAT was controlled for using Western blotting. The protease activity of AuNP-Der p 1 conjugates compared to free Der p 1 was assessed, by an enzymatic activity assay and a cellular assay pertaining to lung type II alveolar epithelial cell tight junction integrity.

**Results:**

The formation of a stable corona was found for all three allergens used. Our data suggest, that depending on the allergen, different effects are observed after binding to ENMs, including enhanced allergic responses against Der p 1 and also, for some patients, against Bet v 1. Moreover elevated protease activity of AuNP-Der p 1 conjugates compared to free Der p 1 was found.

**Conclusion:**

In summary, this study presents that conjugation of allergens to ENMs can modulate the human allergic response, and that protease activity can be increased.

**Graphical Abstract:**
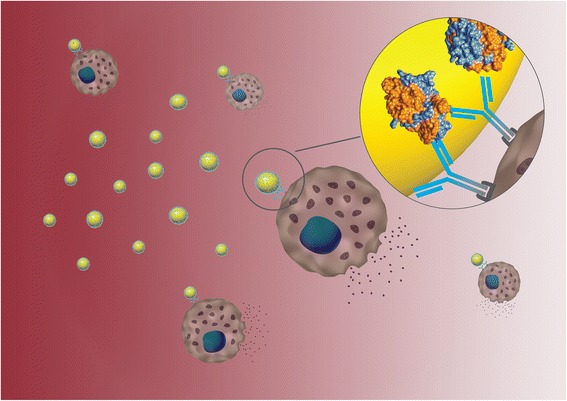
Cross-linking of IgE receptors and degranulation of human basophils due to epitope alignment of nanoparticle-coated allergens.

**Electronic supplementary material:**

The online version of this article (doi:10.1186/s12989-016-0113-0) contains supplementary material, which is available to authorized users.

## Background

With the increasing use of ENMs concerns about the safety upon exposure of workers, consumers and the environment were raised [1–3]. One crucial feature of ENMs is that they possess a higher free energy than the bulk material, which means that the ENMs surface will interact with different biomolecules as soon as they are in contact, resulting in the formation of a ‘corona’ around the ENMs [4]. The biomolecule corona changes the extrinsic properties of the ENMs, but also structures and functions of the biomolecules themselves may be modified upon binding. Thus, if the corona consists of an allergen the overall bio-reactivity of the ENM/corona complex may have the potential to elicit or modulate an allergic response [5–8].

As the overall size of the ENMs would increase only marginally by allergen binding, the ENMs-allergen conjugates could still be inhaled and could translocate from the lung into the bloodstream. Contact with epithelial and/or immune cells and in particular uptake into these cells may result in immune responses [9, 10]. Type I allergy, affecting 30–40 % of the population worldwide, is characterized by the production of immunoglobulin E (IgE) antibodies against allergens. IgE-mediated allergic rhinitis is a risk factor for asthma, a life-long chronic inflammatory disease of the airways, which seriously impacts quality of life and can, when uncontrolled, lead to death [11]. Upon allergic sensitization in the human airways, epithelial cells encountering allergens can release thymic stromal lymphopoietin (TSLP) to attract, activate and polarize dendritic cells (DCs). Activated DCs migrate to lymph nodes, expressing the CC-chemokine ligands (CCL) 17 and CCL22 for T cell attraction [12–14]. In the lymph node, DCs present processed antigens to T cells, and by several mechanisms initiate an immune deviation into a T helper cell 2 (T_H_2)-type profile characterized by secretion of interleukin (IL)-4, IL-5 and IL-13. The T_H_2 cells induce a class-switch in B cells resulting in the production of allergen-specific IgE [15, 16]. During the acute phase of allergy (effector phase), IgE molecules on mast cells and basophils crosslink the cells’ high affinity IgE receptors, initiating a signaling cascade that leads to the release of preformed mediators, enzymes and cytokines, resulting in pathological damage and clinical manifestation of allergy [17–19]. Allergic symptoms can occur seasonally, e.g. after exposure to pollen, or perennially during the exposure to indoor allergens [20]. In Europe the most allergenic tree pollen is produced by birch (major allergen Bet v 1). Pollinosis is also caused by grass pollen, a well-known example being timothy grass (major allergens Phl p 1 and 5) [21]. House dust mite, found in 48 % of European homes, represents the major perennial indoor allergen source (major allergens Der p 1 and 2) [22, 23]. Research on co-exposure to allergens and particles has initially focused on diesel exhaust particles (DEPs). It was shown in previous studies that DEPs can bind Lol p 1, a major grass pollen allergen, and that DEPs can increase the production of allergen-specific IgE in Epstein-Barr virus (EBV)-transformed human B-lymphocytes when exposed to different polycyclic aromatic hydrocarbons (PAHs), a main component found in DEPs [24, 25]. When DEPs and different allergens were administered simultaneously, higher specific IgE levels were found in human nasal lavages as well as in blood drawn from mice [24, 26, 27]. Much less is known about effects of co-exposure of allergens with ENMs. Numerous studies have explored options to employ ENMs as carriers in immunotherapy against allergies [28–32], but the possible risk of unintentional co-exposure has been less explored. The studies performed so far have mainly focused on various established mouse models [33–39].

Furthermore, upon release of ENMs into the environment, which can occur by accident but also upon disposal or via abrasion, ENMs can interact with different allergens. Therefore, this study aimed to determine effects of allergens present as corona compounds on ENMs on the allergic response of human cells, focusing on basophils and on alveolar epithelial cells. Bet v 1 for tree and Phl p 5 for grass pollen, both representing outdoor allergens, and Der p 1, the major allergen for house dust mite, representing indoor allergens were selected. The binding of allergens onto AuNPs and investigated differences in the allergic response of free compared to conjugated allergens was determined. AuNPs were chosen as model ENMs as their reactivity is low, allowing well controlled characterization, and they do not cause an allergic response on their own, enabling an investigation solely on modulation of allergen-specific responses upon particle binding. Furthermore, the behavior of these ENM-allergen conjugates may provide insights into the use of AuNPs as candidates in nanotherapeutic applications. Additionally, as the enzymatic function of Der p 1 as a cysteine protease is considered to play a role in the allergic sensitization, we also investigated the influence of the AuNP-allergen interaction on proteolytic activity of Der p 1 [12, 40].

## Methods

### AuNP-allergen conjugates

AuNPs (3.5*10^10^ NPs/ml, 50 nm, stabilized in 0.1 mM phosphate buffered-saline (PBS), Sigma-Aldrich, St. Louis, MO, USA) were incubated with different concentrations of purified recombinant allergens, i.e. 2.5 μg Bet v 1 (courtesy of C. Ackaert as described in 2014 [41]), 5 μg Der p 1 (Indoor Biotechnologies INC., Cardiff, United Kingdom) or 3 μg Phl p 5 (Allergopharma GmbH, Reinbek, Germany) as described by O. Cromwell et al. [42], on a test tube rotator (Snijders, Tilburg, Netherlands) at 4 °C overnight. The excess protein was removed by performing three washing steps of 5 min centrifugation at 18,000 rpm followed by resuspension in the original buffer.

### Characterization of AuNPs, AuNP-allergen conjugates and allergens

AuNPs were characterized by TEM. The protein corona of AuNP-allergen conjugates was also characterized by TEM with protein negative staining. For these investigations samples were taken directly after the conjugation, before the removal of unbound proteins, and were incubated with 1 % uranyl acetate on coated copper grids to stain the proteins. Images were taken using a LEO 912 AB Omega transmission electron microscope (Carl Zeiss, Oberkochen, Germany) operated with a LaB6 cathode at a voltage of 120 kV. Images were filtered at zero energy loss.

The hydrodynamic radius and surface charge of AuNPs and AuNP-allergen conjugates were determined using a Malvern ZetaSizer Nano ZSP (Malvern Instruments, Malvern, UK). The assessment of the molecular size and potential protein aggregation of the free allergens was performed on the DLS802 (Viscotek, Houston, TX, USA) as previously described by Himly et al. [43].

For further confirmation of hard corona formation, optical and hyperspectral imaging analysis were conducted on a darkfield-based optical illumination system from CytoViva (CytoViva Inc., Auburn, AL, USA). AuNPs and AuNP-allergen conjugates were applied to a microscope slide and covered with a cover glass (both Carl Roth GmbH CoKG, Karlsruhe Germany). The principle of the measurement is based upon the characteristic scattering profile of AuNPs. Each pixel of the image was recorded at a wavelength of 400–1000 nm, and was automatically compared by the classification algorithm spectral angle mapper (SAM) to the AuNP-allergen conjugates. The data is presented as normalized mean regions of interest (ROI’s) of 1100 AuNP-allergen conjugates normalized to the mean ROI of 1100 AuNPs.

### Quantification of the bound allergen

Reversed phase - high performance liquid chromatography (RP-HPLC) was performed to determine the free allergen concentrations before and after the conjugation. Indirect quantification of AuNP-bound allergen was achieved by comparing the obtained peak areas of the UV absorption signals at 214 nm of the original free allergen suspension with the signal obtained from supernatants collected after conjugation with AuNPs, after the above-mentioned washing steps. The measurements were carried out on an UltiMate 3000 system (Thermo-Fisher Scientific Inc., Palo Alto, CA, USA) using a C18 Acclaim^TM^ 300 column (2.1×150 mm, 3 μm, Thermo-Fisher) at a flow rate of 500 μl/min and a column temperature of 50 °C. The gradient of the solvents A (H_2_O + 0.1 % trifluoroacetic acid (TFA)) and B (acetonitrile (ACN) + 0.1 % TFA, both Sigma-Aldrich) was programmed in the following way: 10–60 % solvent A 10 min, 80 % solvent A 5 min, 10 % solvent A 10 min. A full loop injection of 20 μl with a loop volume of 100 μl was used.

### 3D model of AuNP-allergen conjugates

Molecular graphics and analyses were performed with the UCSF Chimera package [44]. Atomic coordinates of allergens were obtained from experimentally determined structures of Bet v 1 (PDB code: 4a88, chain A), Der p 1 (PDB code: 3f5v, chain A) and Phl p 5 (PDB code: 2m64, chain A, NMR model 1) [45, 46]. All three allergens were visualized in a monomeric state and positioned randomly on the surface of an idealized AuNP represented by a sphere of 51 nm diameter, approximately corresponding to the nanoparticle size determined by DLS (see Table 1). Having to decide for plausible orientations of the protein molecules on the AuNP surface, we followed the rationale that the negative potential of a citrate-coated gold surface may select orientations of the protein molecules where positively charged surface patches face the AuNP surface. In order to identify such patches, charges and atom radii were assigned to protein atoms using PDB2PQR [47, 48] and the PARSE parameter set [49]. Subsequently, electrostatic potentials were calculated with the APBS software package [50] (129×129×129 grid points, solute dielectric constant = 2.0, solvent dielectric constant = 78.54, solvent radius = 1.4 Angstrom). The following coarse-grained docking procedure was applied to find electrostatically preferred orientations of a protein molecule relative to the AuNP: for all possible 1×12×12 = 1728 combinations of 30°-rotations about three orthogonal axes centered at the molecule, we minimized the distance of the rotated allergen to the AuNP (distance cutoff = 5 Angstrom) and then summed up the electrostatic potential values at all grid points located at the AuNP surface. Orientations were ranked by this sum and top-ranking solutions (i.e. those with the most positive sum) were chosen for visualization.

**Table 1:** Size distribution, PDI and zeta potential measurements of AuNPs, allergens and AuNP-allergen conjugates

Sample	Average Diameter (nm)	PdI	Difference from AuNPs (nm)	Zeta Potential (mV)	Difference from AuNPs (mV)
Au NPs	50.9 ± 0.6	±0.010	---	- 42.2 ± 2.2	---
Bet v 1	3.92 ± 0.6	---	---	---	---
Der p 1	4.94 ± 2.4	---	---	---	---
Phl p 5	6.48 ± 0.6	---	---	---	---
AuNP-Bet v 1	53.1 ± 1.3	0.101 ± 0.020	2.2 ± 0.7	- 39.2 ± 0.5	3 ± 1.7
AuNP-Der p 1	54.2 ± 2.8	0.141 ± 0.010	3.3 ± 2.2	- 29.6 ± 1.1	12.6 ± 1.1
AuNP-Phl p 5	54.6 ± 1.7	0.103 ± 0.016	3.6 ± 1.1	- 34.2 ± 0.9	8 ± 1.3

### Basophil activation test

Whole blood of nine patients allergic to Bet v 1, five patients allergic to Der p 1 and six patients allergic to Phl p 5, displaying rhinitis and conjunctivitis symptoms, diagnosed at the allergy clinic of the Paracelsus Medical University of Salzburg were studied. The study was approved by the local ethics committee and all patients participating gave their written informed consent. Basophil activation was performed from Ethylene diamine tetraacetic acid (EDTA)-whole blood using Flow CAST^®^ (Buehlmann Laboratories, Schoenenbuch, Switzerland) according to the manufacturer’s protocol. Whole blood samples were stimulated with different concentrations of allergen or AuNP-allergen conjugates (Bet v 1: 50 – 0.048 ng, Der p 1: 50 – 0.04 ng, Phl p 5: 50 – 0.078 ng), and the corresponding amounts of free allergen were included as controls. Processed samples were analyzed by flow cytometry (FACSCanto^TM^ II, BD Bioscience, San Jose, CA, USA) using FACSDiva 5.02. Basophils were gated as side scatter (SSC)^low^/C-C chemokine receptor 3 (CCR3)^high^ and activated basophils were identified as Cluster of differentiation (CD) 63^high^ when cut-off was set with the negative stimulation control. CD63 upregulation was assessed in a minimum of 500 basophils per assay. C50 values (i.e. allergen concentration inducing half-maximal basophil activation) for the AuNP-allergen conjugates were determined by logarithmic approximation (certainty R^2^ > 0.900) in comparison to the allergen alone.

### Enzymatic activity assay

Different concentrations of free Der p 1 (50 and 100 ng) and 30 ng AuNP-Der p 1 conjugates were incubated with 1 mM dithiothreitol (DTT), as reducing agent to activate the cysteine protease, for 10 min at 37 °C. For conjugates the given value refers to the amount of conjugated allergen. After incubation the assay was conducted in 50 mM sodium phosphate buffer, pH 7.0, with 100 μM Boc-Gln-Ala-Arg-AMC (PeptaNova GmbH, Sandhausen, Germany) as substrate according to the manufacturer’s protocol. The fluorescence intensity was recorded at ex/em 380/460 nm using a M200Pro plate reader (Tecan, Groedig, Austria) for 20 min.

### Permeability assay and TEER measurements

The human lung alveolar adenocarcinoma cell line A549 (ATCC, Manassas, USA) was cultured in RPMI 1640 medium (Sigma-Aldrich), supplemented with 10 % fetal calf serum (FCS; PAA, Pasching, Austria), 100 U/ml Penicillin, 100 μg/ml Streptomycin, 2 mM L-glutamine (Sigma-Aldrich). The cells were seeded in 24-well inserts (high pore density, 0.33 cm^2^ growth area, 0.4 μm PET, Merck-Millipore KGaA, Darmstadt, Germany) at a density of 1.5 × 10^5^ cells/ml. Transepithelial electrical resistance (TEER) measurements were performed using a TEER electrode (WPI, Sarasotay, USA) to monitor the cell growth every 24 h. Therefore, the medium was changed and the electrode was washed with RPMI medium between measurements. The TEER values were calculated for the dimension [Ohm*cm^2^] by subtracting the medium only control from the obtained values followed by multiplication with the surface area of the insert. As soon as no further increase in TEER values could be observed, the medium was changed to growth medium without FCS and cells were exposed to 2.5 mM EDTA as a positive control, 0.01 mM DTT, 3.5 × 10^10^ AuNPs, 530 ng free Der p 1, and 3.5 × 10^10^ AuNP-Der p 1 conjugates for 24 h. Thereafter, the medium was changed, and TEER measurements were performed as previously described. For determination of the epithelium permeability A549 cells were incubated with 2.5 μg/insert of Fluorescein (Sigma-Aldrich) at 37 °C for 1 h. To assess the percentage of permeated Fluorescein, 100 μl of the lower compartment was transferred into black culture plates (Greiner Bio-One GmbH, Kremsmuenster, Austria) and the fluorescence at ex/em 485/515 nm was measured using a plate reader (Tecan).

### Statistical analysis

For TEER measurements and permeability experiments, results are expressed as mean value ± standard deviation (SD) of 3 independent experiments; calculated using Microsoft^®^ Office Excel 2007. Statistical analysis was performed using Student’s t-test in GraphPad Prism 5. Due to the donor-to-donor variations expected and observed in the BAT measurements, no statistical analysis was performed for these experiments.

## Results and discussion

### Preparation and characterization of AuNPs and AuNP-allergen conjugates

**Fig. 1:**
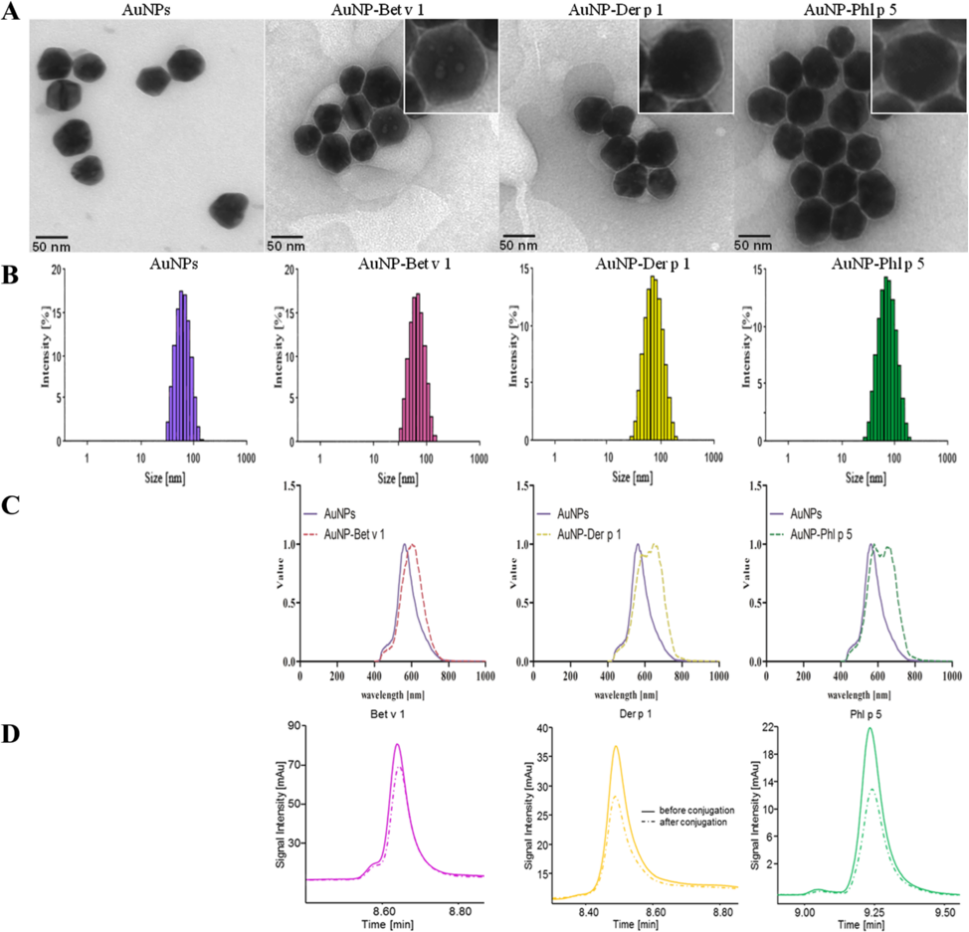
Characterization of AuNPs and AuNP-allergen conjugates. **a** TEM imaging and TEM negative staining. **b** Size distribution by intensity obtained from DLS measurements. **c** Hyperspectral images of AuNPs compared to AuNP-allergen conjugates. **d** HPLC analysis of allergens before and after the conjugation with AuNPs

Allergen conjugation was performed using commercially available citrate-stabilized 50 nm AuNPs (Sigma-Aldrich), and characterized using TEM, DLS, and zeta potential determination. AuNPs were determined to have a negative zeta potential, which was due to the citrate coating to prevent AuNP aggregation (Table 1), and as depicted in Fig. 1a the TEM images show monodispersed AuNPs of approximately 50 nm, confirmed in DLS measurements with a low polydispersity index (PdI) (Fig. 1b). In order to allow the formation of a stable corona, the AuNPs were conjugated overnight with highly purified recombinant allergens, either Bet v 1, Der p 1 or Phl p 5, and the resulting AuNP-allergen conjugates were separated from the excess allergens by a series of centrifugation and washing steps. After the conjugation the presence of an intact protein corona on the AuNP-allergen conjugates was verified using TEM with negative staining (Fig. 1a), DLS (Fig. 1b), zeta potential measurements (Table 1), hyperspectral imaging (Fig. 1c) and RP-HPLC (Fig. 1d).

The TEM negative staining visualized the bound allergen corona as a light halo around the AuNPs on a dark protein background, which was found for all three AuNP-allergen conjugates. Additionally, optical and hyperspectral imaging of the AuNPs and AuNP-allergen conjugates were performed using the system of Cytoviva. A clear shift in wavelength upon allergen binding to AuNPs was found for all three AuNP-allergen conjugates. Results from all methods used for characterization corresponded well and demonstrated that the allergens were successfully conjugated to the AuNPs. Accordingly, the DLS measurements indicated a size increase and zeta potential experiments determined a marked drop in the negative values when comparing AuNP-allergen conjugates to the AuNPs alone. The obtained size increase of the AuNP-allergen conjugates did not correlate with the measured hydrodynamic radius of the allergens, which is due to the fact that DLS gives the hydrodynamic radius of a perfectly shaped sphere, which may often lead to an underestimation of the actual size [51]. Moreover, when investigating the shape of the allergens from their crystal structures as described by de Halleux et al. [52] and Kofler et al. [46] it can be seen that the allergens are not spherical, but rather adopt a cylindrical shape. For quantification of bound allergen we first employed a theoretical determination. For such estimations the equation previously published by Dell’Ocro et al. [53] was used. Therefore, the obtained hydrodynamic radii of the AuNP-allergen conjugates were divided by the hemispheres of the allergens resulting in the maximal numbers of binding sites available for each allergen on the AuNP surface. These calculations showed that DLS data can be used to determine the available binding sites of allergens on the AuNPs surface (Table 2). In order to address this issue experimentally, an indirect quantification using RP-HPLC was conducted (Fig. 1d). Single peaks were detected for the corresponding allergens, which eluted at 8.6 min (Bet v 1), 8.4 min (Der p 1), and 9.2 min (Phl p 5), respectively. The peak areas of allergens before and after the conjugation were compared and the respective allergen concentrations as well as the number of allergen molecules bound to the AuNPs were calculated for each preparation (Table 2). It is noteworthy that the results obtained from both methods matched well, however, for all further experiments the values obtained by the RP-HPLC-based indirect quantification method was used [54].

**Table 2:** DLS and RP-HPLC measurements to determine the amount of conjugated allergen

Sample	Calculated concentration from DLS (ng/ml)	Allergen molecules/AuNP determined by DLS	HPLC determined concentration (ng/ml)	Allergen molecules/AuNP determined by HPLC
AuNP-Bet v 1 conjugates	657 ± 13.3	648 ± 16	490 ± 68.3	516 ± 77
AuNP-Der p 1 conjugates	578 ± 11.7	415 ± 10	530 ± 3.78	379 ± 2
AuNP-Phl p 5 conjugates	365 ± 7.4	224 ± 5	250 ± 24.2	138 ± 13

**Fig. 2:**
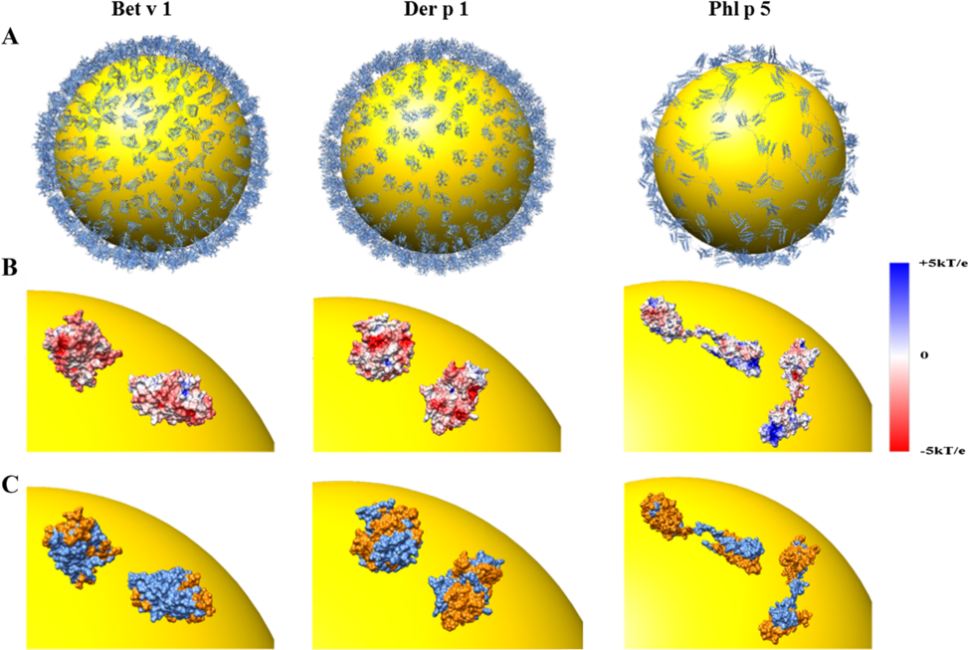
3D models of AuNP-allergen conjugates. **a** Conjugation degree of allergen molecules on a single AuNP. **b** Preferred orientations arrangements of allergen molecules as determined by modelling the interaction with the AuNP surface via electrostatic forces (from +5 kT/e in blue to -5 kT/e in red). **c** Currently known allergen epitopes (*in orange*) accessible for biologic reactions, based on modelled data

For further characterization of binding properties we assessed arrangement and orientation, and more specifically the conjugation degree, of the three AuNP-allergen conjugates [55, 56]. Based on the HPLC results we computed 3D models of AuNP-allergen conjugates (Fig. 2). Approximate numbers of allergen molecules per AuNP were adopted from HPLC experiments (Table 2) and set to 510 (Bet v 1), 380 (Der p 1) and 140 (Phl p 5), illustrating the amount of AuNP surface covered by allergen (Fig. 2a). Figure 2b shows two possible orientations of the three allergens on the AuNP surface. It was assumed that the orientation selection of allergen molecules relative to the citrate-coated AuNP surface is primarily controlled by long-range electrostatic interactions [55, 57, 58]. Therefore, the chosen orientations minimize contacts between negative charges and mainly place positively charged allergen residues on the AuNP surface. In Fig. 2c, these orientations are shown with accessible allergen epitopes highlighted in orange. In all three allergens used the epitopes are available for recognition via IgE.

### Impact of the conjugation to AuNPs on the effector function of allergens

**Fig. 3:**
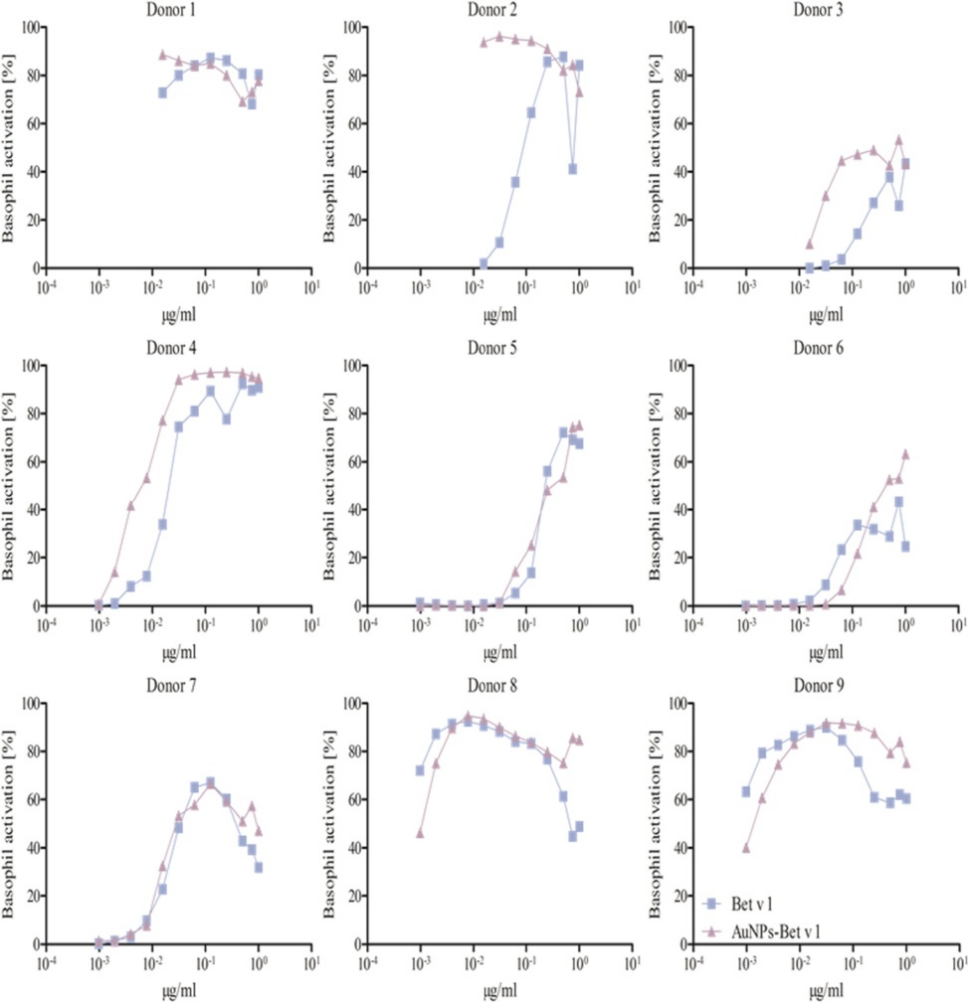
BAT of allergic patients against Bet v 1. The experiments were performed with 9 donors. The patients’ whole blood was treated with 0.048-50 ng free Bet v 1 (*blue squares*) or the respective amount of AuNP-Bet v 1 conjugates (*violet triangles*). Donor 2, 3, 4 and 7 showed a higher activation upon stimulation with AuNP-Bet v 1 conjugates, whereas Donor 5, 6 and 9 were less activated by the AuNP-Bet v 1 conjugates. Donor 8 displayed no difference in the activation pattern. Donor 1 was not used for the determination of the C50 value

**Fig. 4:**
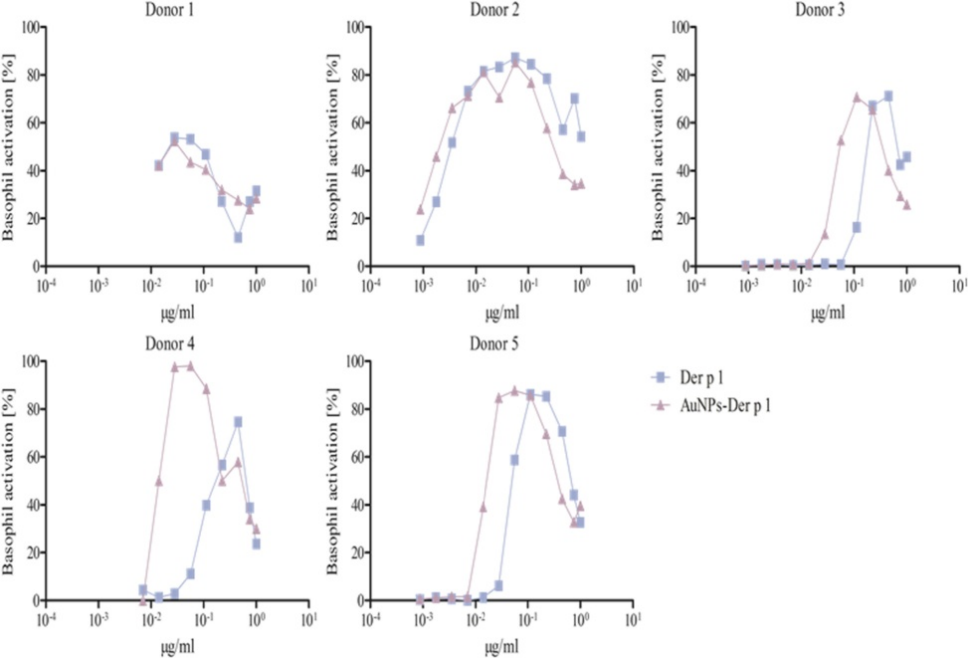
BAT of allergic patients against Der p 1. The experiments were performed with 5 donors. The patients’ whole blood was treated with 0.04-50 ng free Der p 1 (*blue squares*) or the respective amount of AuNP-Der p 1 conjugates (*violet triangles*). Donor 2, 3, 4 and 5 were higher activated by the AuNP-Der p 1 conjugates compared to the allergen alone. Donor 1 was not used for the determination of the C50 value

**Fig. 5:**
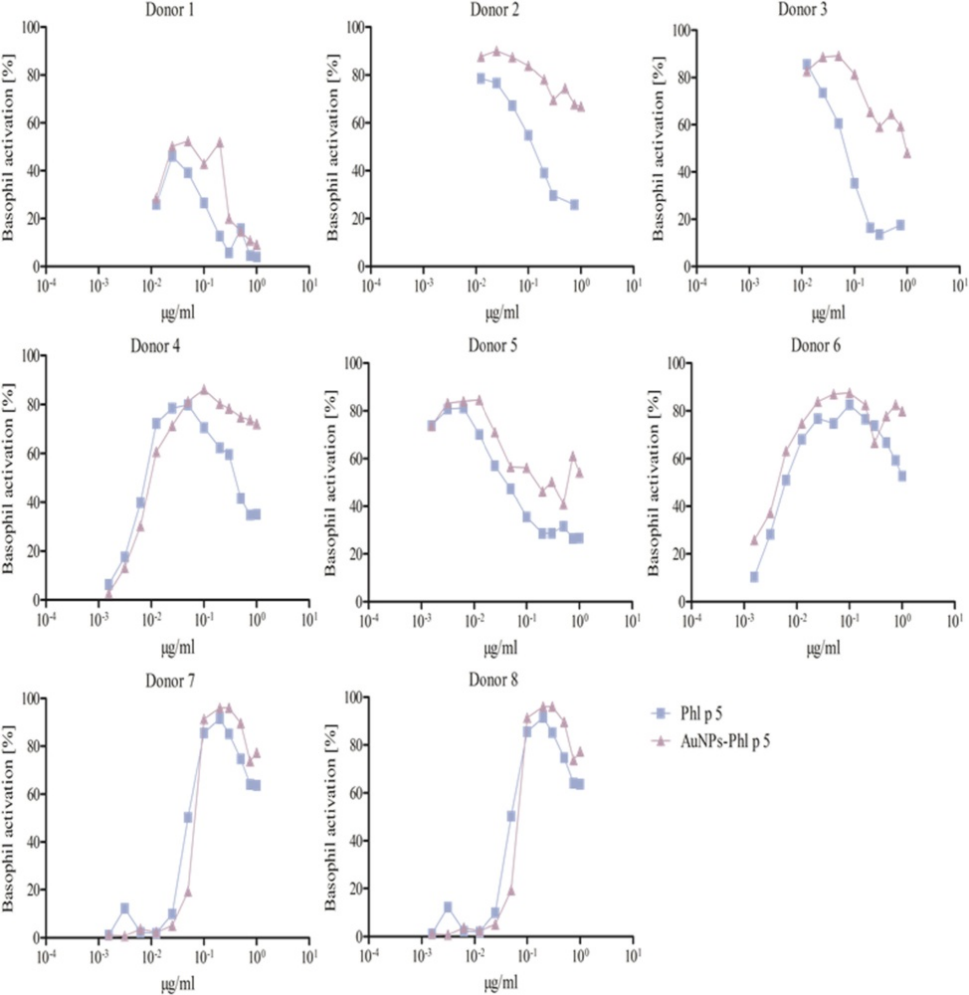
BAT of allergic patients against Phl p 5. The experiments were performed with 8 donors. The patients’ whole blood was treated with 0.078-50 ng free Phl p 5 (*blue squares*) or the respective amount of AuNP-Phl p 5 conjugates (*violet triangles*). Donor 8 showed a higher activation upon stimulation with AuNP-Phl p 5 conjugates, whereas donor 9 was activated to a lesser degree. Donor 4 and 6 did not show a difference in their activation profile. Donor 1, 2, 3 and 5 were not used for the determination of the C50 value

After using various methods for protein corona characterization, we were confident that AuNP-allergen conjugates had been formed. Other methods are available, such as nuclear magnetic resonance, infrared spectroscopy or circular dichroism, which provide an indication of structural conformation [56]. The structural integrity of the allergens used in this study when bound to AuNPs is important since structural changes can modulate the biological response. However, the use of these methods by Calzolai et al. [56] require >400-fold more protein than we used in our study, making these methods impossible to use in our experimental conditions. Furthermore, we consider that allergen-specific cellular responses are mainly driven by the availability and accessibility of intact epitopes, which are not limited to a particular tertiary structure or orientation of the allergen. Therefore, we performed the BAT, as its clinical relevance as a highly specific in vitro test system for allergenic activity was just recently emphasized by a position paper of the European Academy for Allergy and Clinical Immunology by Hoffmann et al., in 2015 including the use of BAT to monitor the patient’s sensitivity to inhalant allergens over time [59]. The aim was to provide not only an indication of available epitopes, but also to determine if the binding of allergens to AuNPs can heighten or reduce an allergic reaction. This test gives a direct assessment of allergenic effects in sensitized humans. Although human basophils are the least abundant population of granulocytes, they play a crucial role in the immediate-type allergic reaction and moreover in allergic inflammation in general [17, 60, 61]. The exposure of basophils, from sensitized subjects, to allergens induces cross-linking of the allergen-specific IgE bound to the cell surface via its high affinity receptor for IgE (FcεRI), facilitated by the presence of multiple non-overlapping IgE-binding epitopes, resulting in cell degranulation with the release of preformed mediator substances including histamine, leukotrienes, prostaglandins, and proteases. Upon degranulation, the basophil activation marker CD63, which in resting basophils is located only in the membrane of the granules, is expressed at the surface of the basophils, and this correlates with the released histamine concentration [62–64]. Donor-to-donor variations can occur, as not only the amount of allergen-specific IgE in the serum and the number of FcεRI can vary, but also the IgE repertoire can be different between different patients. This is expected for a natural polyclonal IgE response, as for a number of major allergens a distribution of IgE epitopes across the entire surface has been shown [62, 65, 66]. Accordingly, a number of conformational, i.e. discontinuous IgE epitopes of Bet v 1, Der p 1 and Phl p 5 have been identified by mAB-based, epitope grafting, site-directed mutagenesis, X-ray crystallography and mimotope approaches [45, 65, 67–73]. Our 3D model of AuNP-allergen conjugates hypothesizes coupling of the allergens to the citrate-coated AuNP surface based on electrostatic interaction, which may, in the case of certain examples of positively charged interaction patches, result in an alignment on the AuNP surface where the allergens repetitively display the same epitopes. To investigate integrity and accessibility of IgE epitopes, experiments were performed using allergic patients’ whole blood and stimulation of the basophils with the allergen, the AuNP-allergen conjugates and the plain AuNPs. No basophil activation was found when exposed to the AuNPs alone. The assay was performed for nine donors allergic to Bet v 1, five donors allergic to Der p 1, and eight donors allergic to Phl p 5. All donors tested could be divided into three categories:

i. Donors that did not display a difference in basophil activation when comparing free versus AuNP-allergen conjugates, as similar C50 values were observed. These included examples from each patient subset, with two, and three examples for Bet v 1 and Phl p 5, respectively (Figs. 3, 4, 5, Table 3). This infers that in these donors the allergen-specific IgE antibodies recognized the same epitopes irrespective of whether the allergens were free or conjugated to the AuNPs.
ii. Donors with lower basophil activation and higher C50 values upon stimulation with AuNP-allergen conjugates compared to free allergen, as shown for Bet v 1 and Phl p 5, with three and one examples, respectively (Figs. 3, 5, Table 3). In the case of Der p 1, no reduction in basophil activation was found when allergen was bound to the AuNPs. This observation may be explained by an allergen arrangement on the AuNPs surface, where epitopes are partially hidden or that these patients’ do not display the specific IgE against this epitope [62, 74].
iii. Donors which displayed an increase in basophil activation and thus lower C50 values when cells were exposed to the AuNP-allergen conjugates compared to free allergen. This was shown in three Bet v 1 donors, four Der p 1 donors, and one Phl p 5 donor (Figs. 3, 4, 5, Table 3). With our hypothesized uniform binding orientation, the conjugated allergens would align in the same direction enabling more IgE molecules to crosslink through an increased localized concentration of the bound allergen [75].

**Table 3:** Concentrations of half-maximal basophil activation (C50) of free allergen versus AuNP-allergen conjugates for all donors tested. In conjugates the given values refer to the amounts of coupled allergen. Donors displaying X were tested but could not be statistically evaluated

	C50 [ng/ml]					
Donor	Bet v 1	AuNP-Bet v 1 conjugates	Der p 1	AuNP-Der p 1 conjugates	Phl p 5	AuNP-Phl p 5 conjugates
1	X	X	X	X	X	X
2	178	1	4	2	X	X
3	444	51	186	55	X	X
4	52	13	27	1	16	19
5	287	548	58	20	X	X
6	78	107	---	---	1	1
7	48	43	---	---	29	12
8	2	2	---	---	65	77
9	12	89	---	---	---	---

It was furthermore observed that four donors, two for Bet v 1 (donors 3, 6), one for Der p 1 (donor 1), and one for Phl p 5 (donor 1), displayed a lower basophil sensitivity which manifested in a lower maximal activation response (Figs. 3, 4 and 5). Such examples have been described before and may be explained by either a lower total IgE concentration or less allergen-specific IgE bound to the basophils in those patients compared to the others [62].

### Using the enzymatic function for studying protein-nanoparticle interactions – the protease activity of Der p 1

**Fig. 6:**
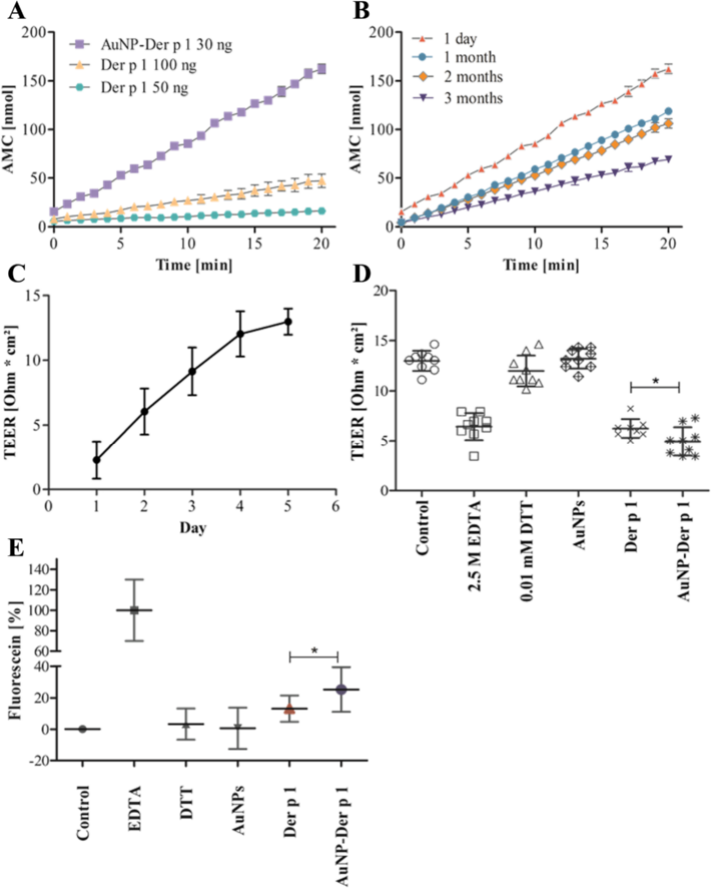
Determination of protease activity of free Der p 1 versus AuNP-Der p 1 conjugates. **a** Enzymatic activity assay of 30 ng AuNP-Der p 1 conjugates compared to 50 and 100 ng free Der p 1. **b** Stability of conjugates addressed by enzymatic activity of 30 ng AuNP-Der p 1 conjugates upon storage at 4 °C. In conjugates the given values refer to amount of coupled allergen. **c** TEER measurements of A549 cells to monitor the formation of a tight cell layer. **d** TEER measurements of A549 cells exposed to controls, free Der p 1 and AuNP-Der p 1 conjugates (*, *p* < 0.05). **e** Permeation of Fluorescein through a tight monolayer of A549 cells exposed to controls, free Der p 1 and AuNP-Der p 1 conjugates (*, *p* < 0.05)

Since Der p 1 is known to be a cysteine protease [76], this intrinsic property was used to investigate the activity of free Der p 1 versus AuNP-Der p 1 conjugates in an enzyme activity assay and, secondly, their effect on cell monolayer tight junctions. For the enzyme activity assay, the effect of Der p 1 on t-Butyloxycarbonyl-L-glutaminyl-L-alanyl-L-arginine-4-methylcourmaryl-z-amide (Boc-Gln-Ala-Arg-AMC) was investigated. Figure 6a displays the results obtained with 100 ng and 50 ng free Der p 1 compared to 30 ng of conjugated Der p 1. After 20 min the AMC release was 3-fold higher in case for AuNP-Der p 1 conjugates compared to 100 ng Der p 1 and 8-fold higher compared to 50 ng Der p 1. After 40 min the difference in AMC release was still 2-fold higher than for 100 ng and 5-fold higher than for 50 ng Der p 1 (data not shown). This observation could be due to the alignment of Der p 1 on the AuNP surface, which could lead to the observed higher enzymatic activity.

Additionally, the stability of conjugates, and enzyme activity, upon storage at 4 °C was investigated. Hence, the AuNP-Der p 1 conjugates were analyzed after one, two and three months of storage and compared to the fresh AuNP-Der p 1 conjugates. In Fig. 6b the difference in AMC release for AuNP-Der p 1 conjugates is shown; with a decrease in AMC release of 1.3-fold, 1.5-fold and 2.2-fold after one, two and three months, respectively, when compared to the fresh conjugates. These results gave an insight in the stability of the protein corona of Der p 1 conjugated to AuNPs. Upon storage in 0.1 M PBS without an excess of the protein, the proteins either slowly start to detach from the AuNPs surface or are subject to a limited degree of degradation.

The protease function of Der p 1 has been reported to play a functional role in allergic sensitization, as the protease function may disrupt the epithelial barrier in the lung [40, 76]. Consequently, such a marked increase in the enzymatic activity of AuNP-Der p 1 conjugates may have strong implications for allergic sensitization and lung pathophysiology upon uptake via inhalation. Therefore, permeability assays in A549 cells were carried out to address whether in a more physiological model, compared to the enzyme activity assay, such a high impact of AuNPs-Der p 1 conjugates could be found. A549 are cancer cells derived from human type II alveolar epithelial cells, frequently used in investigating effects of inhaled ENMs, since they can form tight epithelia and reproduce other features of this cell type, for which primary cells cannot be obtained. Figure 6c displays the TEER measurements, as a measure of barrier function, of A549 cells grown on the membrane of an insert until forming a tight cell layer. The TEER values of the control cells on day 4 and 5 did not show significant further increases, indicating the formation of a tight monolayer at this time point, as previously described by Schlinkert et al. [77]. On day 4 the cells were treated for 24 h with free Der p 1, AuNP-Der p 1 conjugates, which were activated with 0.1 mM DTT prior to the exposure, 2.5 M EDTA as a positive control, DTT and plain AuNPs, to determine if any would alter the TEER values, or left untreated (Fig. 6d). The EDTA positive control, and more noteworthy, both allergen preparations, free Der p 1 and AuNP-Der p 1 conjugates significantly decreased barrier function of the lung epithelial cells. Furthermore, when comparing free Der p 1 to AuNP-Der p1 conjugates, AuNP-Der p 1 conjugates had a higher impact on the integrity of the monolayer (*P* < 0.05). In contrast, neither 0.01 mM DTT (used for activation of the protease function) nor the AuNPs alone had any effect on TEER values. The concentration of fluorescein permeating the tight layer was investigated using the same treatments as described above (Fig. 6e) [78]. These data were in line with the TEER measurements, with a significant increase in permeation when cells were exposed to AuNP-Der p 1 conjugates (*P* < 0.05) compared to the same amount of free Der p 1. Both experiments verified the significant impact on the cell layer when cells were exposed to AuNP-Der p 1 conjugates, which also correlated well with the data observed in the enzymatic activity assay.

## Conclusions

In this study we have shown the successful conjugation of three major outdoor and indoor allergens (Bet v 1, Der p 1 and Phl p 5) as highly purified and well-characterized recombinant molecules to 50 nm AuNPs. The AuNP-allergen conjugates were characterized by TEM negative staining, DLS and hyperspectral imaging. All three characterization methods gave correlating results, namely that the allergens were firmly interacting with the AuNPs’ surface. Indirect quantification by RP-HPLC was performed allowing the determination of the allergen concentration on the AuNPs’ surface. To visualize the interaction between the allergens and the AuNPs at the molecular level a 3D model was established based on crystal and NMR structural information, depicting the arrangement of the allergens on the AuNPs upon electrostatic interaction and the surface exposure of the known IgE-binding epitopes of the allergens. For providing experimental evidence for the generated models, the integrity and accessibility of IgE epitopes was determined by activation assays using human blood basophils derived from a panel of allergic patients. We observed a high donor-to-donor variability, depicting similar, higher or lower basophil responses to free allergen versus the respective amounts of AuNP-allergen conjugates. This led us to the conclusion that, (i) in case of similar results, the IgE epitopes of the allergens can be recognized equally well, (ii) in case of a lower basophil activation, some epitopes may become hidden, and the patients’ specific IgE antibodies are not able to recognize other IgE epitopes present on the allergens’ surface, and (iii) in the case of higher basophil activation, an alignment of the allergens on the AuNPs’ surface takes place which optimally displays the IgE epitopes relevant for the respective patient. This would facilitate a more effective IgE receptor crosslinking on the basophils due to a higher localized concentration of allergens on the AuNPs compared to the free allergen.

AuNP-Der p 1 conjugates were the only AuNP-allergen conjugates affected by replacement of plasma proteins, when determining the “hardness” of the protein corona formed via Western blots as control experiments for the AuNP-allergen conjugate incubation in plasma during BAT (Additional File 1: Supplementary material and Additional File 2: Figure S1). We, furthermore, determined that the enzymatic activity of Der p 1 upon binding to AuNPs was markedly enhanced in a cell free assay, and accordingly, the conjugates reduced the integrity of a tight cell monolayer when compared to free Der p 1. Release of Der p 1 from AuNPs was not fast enough, or to a high enough level, to abolish an allergy-promoting effect as shown by increased basophil activation. Studies with other allergens are needed to show how frequent such effects are for allergens in general.

In summary, this study presents that conjugation of allergens to ENMs can modulate the allergic response. Yet, due to allergic donor variability, this effect was not consistently observed. Furthermore the enzymatic activity of the protease Der p 1 was increased when conjugated to ENMs.

## Additional files

Additional file 1 **Figure S1.** Supplementary material. Control for allergen corona replacement during basophil activation assays. (DOCX 573 kb)

Additional file 2 Determination of conjugated allergen after the incubation in human plasma performed by Coomassie-stained SDS-PAGE (A-C) and Western blots (D-F). (TIF 526 kb)

## Abbreviations

ACN: acetonitrile
AuNPs: gold nanoparticles
BAT: basophil activation test
Boc-Gln-Ala-Arg-AMC: t-Butyloxycarbonyl-L-glutaminyl-L-alanyl-L-arginine-4-methylcourmaryl-z-amide
CCL: CC-chemokine ligand
CCR: CC-chemokine receptor
CD63: cluster of differentiation 63
DCs: dendritic cells
DEPs: diesel exhaust particles
DLS: dynamic light scattering
DTT: dithiothreitol
EDTA: ethylene diamine tetraacetic acid
ENMs: engineered nanomaterials
FCS: fetal calf serum
FcεRI: high affinity receptor for IgE
HRP: horse radish peroxidase
Ig: immunoglobulin
IL: interleukin
mAb: monoclonal antibody
NMR: nuclear magnetic resonance
PBS: phosphate-buffered saline
PdI: polydispersity index
ROI: region of interest
RP-HPLC: reversed phase-high performance liquid chromatography
SAM: spectral angle mapper
SD: standard deviation
SDS: sodium dodecyl sulfate
SDS-PAGE: sodium dodecyl sulfate polyacrylamide gel
SSC: side scatter
TBS: tris-buffered saline
TEER: transepithelial electrical resistance
TEM: transmission electron microscopy
TFA: trifluoroacetic acid
T_H_2: T helper cell 2
TSLP: thymic stromal lymphopoietin
