# Supplementary figures and images for: Nanoparticle-allergen interactions mediate human allergic responses: protein corona characterization and cellular responses
Source: Part Fibre Toxicol. 2016 Jan 16;13:3. doi: 10.1186/s12989-016-0113-0 (PMC4715273; doi:10.1186/s12989-016-0113-0)

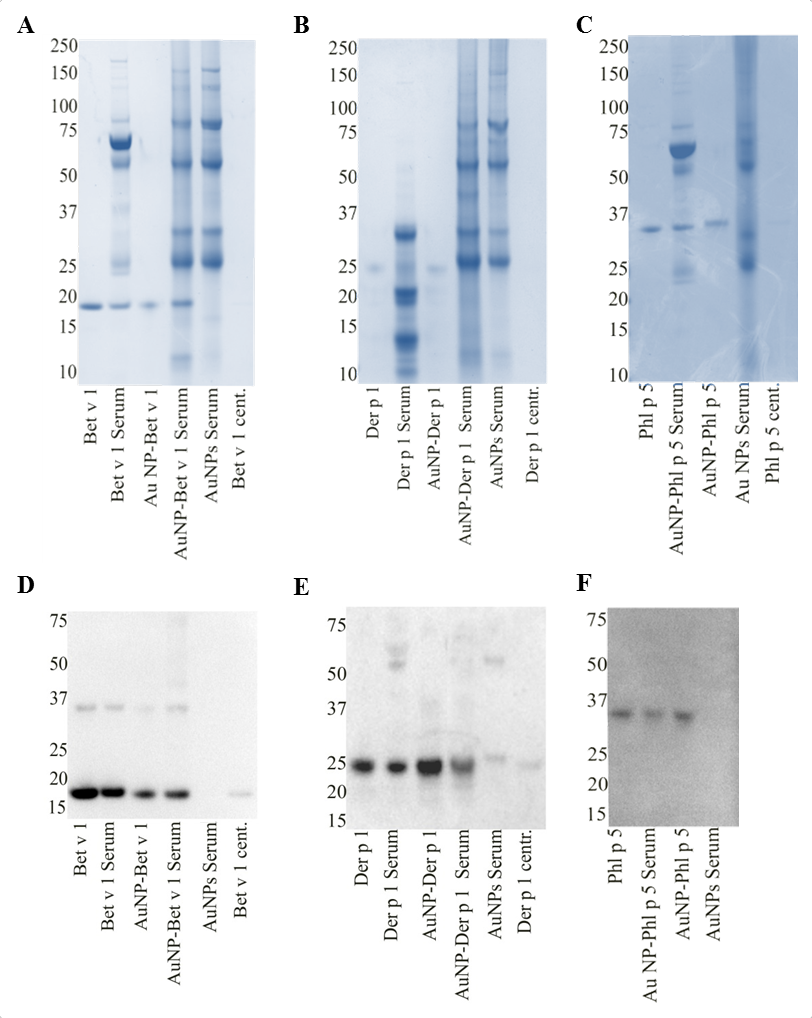

Supplement: Supplementary file 1 — Figure S1. Supplementary material. Control for allergen corona replacement during basophil activation assays. (DOCX 573 kb) [file 12989_2016_113_MOESM1_ESM.tif]
