# Supplementary material for: Nanoparticle-allergen interactions mediate human allergic responses: protein corona characterization and cellular responses
Source: Part Fibre Toxicol. 2016 Jan 16;13:3. doi: 10.1186/s12989-016-0113-0 (PMC4715273; doi:10.1186/s12989-016-0113-0)
Supplement: Supplementary file 2 — Determination of conjugated allergen after the incubation in human plasma performed by Coomassie-stained SDS-PAGE (A-C) and Western blots (D-F). (TIF 526 kb) [file 12989_2016_113_MOESM2_ESM.docx]

**Supplementary Material**

Nanoparticle–allergen interactions mediate human allergic responses: protein corona characterization and cellular responses

Isabella Radauer-Preiml, Ancuela Andosch, Thomas Hawranek, Ursula Luetz-Meindl, Markus Wiederstein, Jutta Horejs-Hoeck, Martin Himly, Matthew Boyles, Albert Duschl

**MATERIALS and METHODS**

**SDS-PAGE and Western blotting**

The composition of the hard corona after incubation in human serum was investigated using sodium dodecyl sulfate polyacrylamide gel electrophoresis (SDS-PAGE) and Western blotting experiments. Therefore, the free allergens and AuNP-allergen conjugates were incubated in 13% of human plasma derived from a non-allergic/asymptomatic blood donor (to circumvent interaction of allergen-specific IgE with the allergen corona) at 37°C for 15 min. Thereafter, the AuNP-allergen conjugates were washed thrice as described above. The samples were denatured in 2x Laemmli sample buffer (Bio-Rad, Vienna, Austria) at 95°C for 20 min. The samples were loaded on precast 4-12% gradient gels (NuPAGE, Invitrogen, Lofer, Austria) and either stained with Coomassie Brilliant blue R-250, or blotted onto nitrocellulose membrane (both Bio-Rad). The membrane was blocked with Tris-buffered saline (TBS) containing 0.1% Tween 20 and 5% non-fat dry milk at room temperature for 1 hour. The primary antibodies (Bet v 1: mouse, IgG1, Phl p 5: human, IgG1, Der p 1: polyclonal rabbit antiserum) were produced as previously described [[1-3](#_ENREF_1)]. IgG was purified from monoclonal antibody (mAb)-containing hybridoma supernatants and from rabbit serum using recombinant protein G-Sepharose 4B Conjugate (Invitrogen Corporation, CA, USA). Optimal specificity and sensitivity was found at dilutions of 1:10,000 (Bet v 1), 1:1,000 (Der p 1), and 1:50 (Phl p 5). Horse radish peroxidase (HRP)-linked secondary Abs (Cell Signaling Technology, MA, USA) were used at a dilution of 1:1,000. Detection was performed using Supersignal ECL substrate (West Pico, Pierce, IL, USA), for imaging of gels and Western blots a ChemiDoc MP Imaging system with Image Lab Software (Bio-Rad) was used.

**RESULTS and DISCUSSION**

**Control for allergen corona replacement during basophil activation assays**

It is well known that ENMs become coated with proteins upon contact with complex biological fluids, and this process may be dynamic upon change of the respective biological environment [[4-6](#_ENREF_4)]. As the basophil activation test (BAT) required incubation of the AuNP-allergen conjugates in human plasma containing high concentrations of serum proteins, replacement of the allergens from the corona during BAT needed to be monitored. In this study the “hard” corona of the AuNP-allergen conjugates consisted of only one type of protein per conjugate, the respective allergen. Therefore the stability of this corona after the incubation in human plasma was addressed. This enabled us to obtain insights into the interaction of the AuNP-allergen conjugates with human whole blood, as this was used in the basophil activation experiments. Therefore, AuNP-allergen conjugates were incubated in human plasma for 15 min followed by several washing steps to remove the unbound fraction. The bound allergens and other serum proteins in the corona were removed from the AuNPs by heat denaturation and binding to SDS and separated by SDS-PAGE (Figure S1). With Coomassie staining we identified that the allergen bands obtained from the AuNP-Bet v 1 and -Phl p 5 conjugates did not decrease in intensity when incubated in plasma compared to the same concentration of free allergen incubated accordingly concluding that the exchange of allergens from the corona was limited in the time frame of BAT. For AuNP-Der p 1 conjugates incubated in plasma, the Der p 1 band was not clearly identified in the Coomassie-stained gel, as it could not be resolved from another plasma protein band with 26 kD (Figure S1B). Moreover, it became evident from the Coomassie-stained gels that a number of plasma proteins were interacting with the AuNP-allergen conjugates during functional tests involving human blood. As much work on the interaction of NPs with human plasma has previously been done, we were able to identify the interacting plasma proteins based on the literature on the protein corona of gold, polystyrene, silica, and copolymer NPs after incubation in plasma (Table S1) [[6-9](#_ENREF_6)].

Only the intensity of the 75 kD (IgM heavy chain) band was found decreased in case of AuNP-Bet v 1 and -Der p 1 conjugates compared to when AuNPs were incubated alone in plasma (Figure S1A and B), while the protein bands at a molecular weight (MW) of 26 kD (apolipoprotein A-I), 30 kD (apolipoprotein E), 51 kD (Ig heavy chain) and 75 kD (IgM heavy chain) were found decreased comparing AuNP-Phl p 5 conjugates with AuNPs alone in plasma. In contrast, a strong increase was found for the band at 69 kD, which was most likely human serum albumin (HSA) (Figure S1C).

In order to further monitor the presence of allergen after incubation in human plasma, and more specifically to determine the allergen corona stability, Western blots were performed (Figure S1D-F). Monomers and dimers of Bet v 1 were recognized by their mAb, before and after incubation in plasma. There was no indication of any unspecific binding, as shown in the lane of AuNPs alone incubated with plasma. However, a small degree of protein adsorption to the sample vials was found, as a faint band was observed in the lane where a solution of Bet v 1 alone was taken through the same washing procedure as the other samples. However, the intensity was much weaker in this lane, compared to the lane of AuNP-Bet v 1 conjugates (Figure S1A). In contrast, AuNP-Der p 1 conjugates incubated in plasma displayed a lower intensity compared to AuNP-Der p 1 conjugates before plasma incubation. Two faint bands were found in the AuNPs control, which were determined to be due to some low degree of unspecific binding of the polyclonal antibody, as the lane for plasma plus AuNPs alone had similar bands (Figure S1B). As was seen for Der p 1, the signal detected for Phl p 5 in AuNP-Phl p 5 conjugates had a slightly lower intensity compared to the AuNP-Phl p 5 conjugates before plasma incubation. No indication of unspecific binding was detectable in the lane where AuNPs only had been incubated with plasma (Figure S1C).

The lower intensity upon plasma incubation that was observed for the AuNP-Der p 1 and -Phl p 5 conjugates could be explained by earlier findings, where it was shown that the protein corona can integrate new proteins or can replace proteins of lower affinity with those of higher affinity as the biological environment changes [[10](#_ENREF_10), [11](#_ENREF_11)]. Since the donor, although also confirmed to be non-allergic to Bet v 1 and Phl p 5, was slightly allergic to Der p 1, the determined decrease of the Der p 1 conjugates (but not the others) incubated in plasma could to some extent be due to the interaction of the donor’s IgE with the conjugated Der p 1, masking the epitopes for the rabbit serum used for the detection. As the protein turnover within a protein corona may occur within minutes, there are particular challenges to be addressed experimentally. However, this study clearly demonstrates that basophil activation is a process running sufficiently fast, and the corona was shown to be relatively stable during this time. Thus, the approach to determine differences in the biological responses to the coupled allergens can be considered valid. This agrees well with the short time frame of type I allergy, as it is well known that the onset of this type of hypersensitivity reactions establishes immediately (within a few minutes). Thus, the allergic effector function represents a promising tool for the characterization of the dynamics of allergen-NP interactions, provided an allergic individual can be found for the respective protein under investigation.


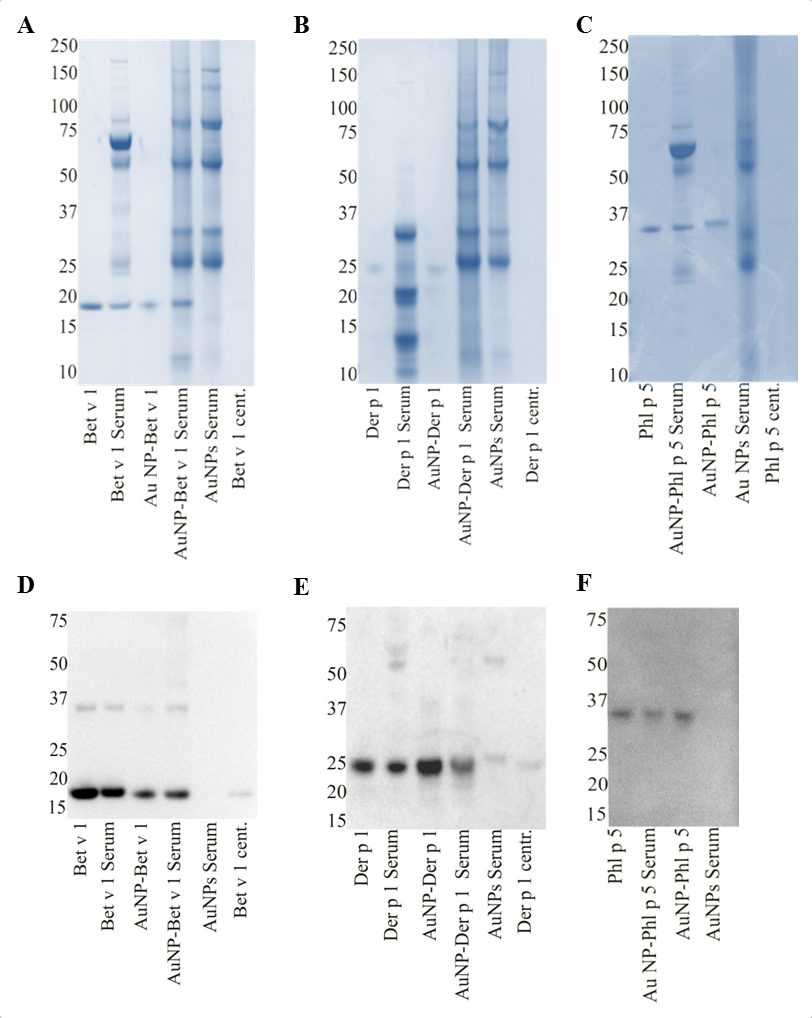


**Figure S1.** Determination of conjugated allergen after the incubation in human plasma performed by Coomassie-stained SDS-PAGE (A-C) and Western blots (D-F).

**Table S1.** Presumptive human plasma proteins interacting with AuNPs and AuNP-allergen conjugates during the incubation, based on SDS-PAGE analysis and molecular weight comparison with values from current literature.

| Plasma proteins interacting with AuNPs | | |
| --- | --- | --- |
| MW  [kD] | Protein | Literature |
| 12 | Ig kappa chain | Cedervall *et al.[*[*7*](#_ENREF_7)*]* |
| 26 | Apolipoprotein A-I | Monopoli *et al.[*[*6*](#_ENREF_6)*]* |
| 30 | Apolipoprotein E | Monopoli *et al.[*[*6*](#_ENREF_6)*]* |
| 51 | Ig heavy chain | Deng *et al.[*[*8*](#_ENREF_8)*]* |
| 69 | HSA | Cedervall *et al.[*[*7*](#_ENREF_7)*]* |
| 75 | IgM heavy chain | Putnam F.[[9](#_ENREF_9)] |

**References**

1. Chen KW, Blatt K, Thomas WR et al. Hypoallergenic Der p 1/Der p 2 combination vaccines for immunotherapy of house dust mite allergy. The Journal of allergy and clinical immunology. 2012;130(2):435-43 e4. doi:10.1016/j.jaci.2012.05.035.

2. Flicker S, Vrtala S, Steinberger P et al. A human monoclonal IgE antibody defines a highly allergenic fragment of the major timothy grass pollen allergen, Phl p 5: molecular, immunological, and structural characterization of the epitope-containing domain. J Immunol. 2000;165(7):3849-59.

3. Laffer S, Vangelista L, Steinberger P et al. Molecular characterization of Bip 1, a monoclonal antibody that modulates IgE binding to birch pollen allergen, Bet v 1. J Immunol. 1996;157(11):4953-62.

4. Lundqvist M, Stigler J, Cedervall T et al. The evolution of the protein corona around nanoparticles: a test study. ACS nano. 2011;5(9):7503-9. doi:10.1021/nn202458g.

5. Lynch I, Dawson KA. Protein-nanoparticle interactions. Nano Today. 2008;3(1):40-7.

6. Monopoli MP, Aberg C, Salvati A et al. Biomolecular coronas provide the biological identity of nanosized materials. Nature nanotechnology. 2012;7(12):779-86. doi:10.1038/nnano.2012.207.

7. Cedervall T, Lynch I, Foy M et al. Detailed identification of plasma proteins adsorbed on copolymer nanoparticles. Angewandte Chemie. 2007;46(30):5754-6. doi:10.1002/anie.200700465.

8. Deng ZJ, Liang M, Toth I et al. Plasma protein binding of positively and negatively charged polymer-coated gold nanoparticles elicits different biological responses. Nanotoxicology. 2013;7:314-22. doi:10.3109/17435390.2012.655342.

9. Putnam F. The plasma proteins. Elsevier; 2012.

10. Casals E, Pfaller T, Duschl A et al. Hardening of the nanoparticle-protein corona in metal (Au, Ag) and oxide (Fe3O4, CoO, and CeO2) nanoparticles. Small. 2011;7(24):3479-86. doi:10.1002/smll.201101511.

11. Lundqvist M, Stigler J, Elia G et al. Nanoparticle size and surface properties determine the protein corona with possible implications for biological impacts. Proceedings of the National Academy of Sciences of the United States of America. 2008;105(38):14265-70. doi:10.1073/pnas.0805135105.
